# Molecular architecture of primate specific neural circuit formation

**DOI:** 10.21203/rs.3.rs-4082064/v1

**Published:** 2024-03-22

**Authors:** Tomomi Shimogori, Kohei Onishi, Takafumi Hoshino, Moe Nakanishi

**Affiliations:** Molecular Mechanisms of Brain Development, Center for Brain Science (CBS), RIKEN; Molecular Mechanisms of Brain Development, Center for Brain Science (CBS), RIKEN; Center for Brain Science, RIKEN; Center for Brain Science, RIKEN

## Abstract

The mammalian cortex is a highly evolved brain region, but we still lack a comprehensive understanding of the molecular mechanisms underlying primate-specific neural circuits formation. In this study, we employed spatial transcriptomics to assess gene expression dynamics in the marmoset cortex during development, focusing on key regions and time points. Spatial transcriptomics identified genes that are sexually, spatially, and temporally differentially expressed in the developing marmoset cortex. Our detailed analysis of the visual cortex unveiled dynamic changes in gene expression across layers with distinct projections and functions. Notably, we discovered numerous axon guidance molecules with spatiotemporal expression patterns unique to the developing marmoset prefrontal cortex (PFC), which control PFC neuronal circuits. Among these molecules, *PRSS12* (Protease, Serine, 12 (neurotrypsin, motopsin), when ectopically expressed in the mouse prelimbic cortex, caused similar changes in connectivity as observed in the marmoset A32 area. Furthermore, *PRSS12* showed similar expression patterns in both marmoset and human PFC during development, suggesting parallels between marmoset and human brain development. The differential expression of axon guidance molecules in the developing PFC, varying by region, likely contributes to the formation of unique circuits observed in primates.

## Introduction

The cerebral cortex, responsible for higher cognitive functions, including the primate-specific prefrontal cortex, is crucial in evolution^1^. It is believed that the evolution of the cerebral cortex is driven by multiple mechanisms, including gene mutations, selection pressures, and changes in developmental processess^2^. However, the precise mechanisms involved are still not fully understood. Understanding its development requires analyzing gene expression patterns across mammalian species. While rodent studies offer insight, studying primate cortical development, especially in species like the common marmoset, provides valuable clues due to their brain’s similarity to humans^3–10^. Our analysis of gene expression by spatial transcriptomics, in various neocortical regions during marmoset development revealed time-specific, region-specific cortical layer-specific gene expression. We hypothesized that this differential expression, especially of axon guidance factors in the PFC, could reveal molecular processes governing evolutionary diversification. Our functional gene expression analysis provide evidence that these species-specific spatiotemporally expressed genes play important role to generate species-specific neuronal circuit.

## Result

### Sample preparation and analysis for 10x spatial transcriptomics

**Figure 1:**
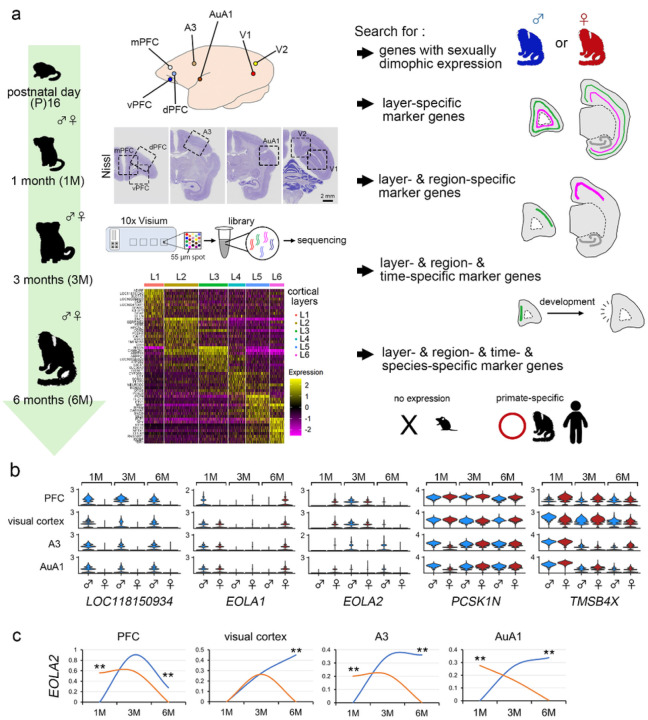
Sexually dimorphic gene expression in the marmoset developing cortex (a) Sample preparation from developing marmosets for 10x Visium analysis. Cortical areas from medial prefrontal cortex (mPFC), ventral PFC (vPFC), dorsal PFC (dPFC), A3, AuA1, V1, and V2 were analyzed. The areas analyzed for mPFC, vPFC, dPFC, A3, AuA1, V1, and V2, enclosed by a black dashed square, were shown in the images of Nissl staining of the 1-month marmoset brain. The brains were collected from postnatal day (P) 16, 1-month (1M), 3-months (3M), and 6-months (6M) marmosets. The target region from a fresh frozen section of the marmoset cortex and placing it on a 6.5 × 6.5 mm 10x Visium platform. The heat map displays markers across cortical layer cell populations, presenting the expression of the top 10 genes in the 1M AuA1 cortical layers. (b) Violin plots indicate gene expression levels for *LOC118150934, EOLA1, EOLA2, PCSK1N,* and *TMSB4X* in each region (PFC, visual cortex, A3, and AuA1) for males and females at 1M, 3M, and 6M. (c)Trends in gene expression are depicted as graphs for each region (PFC, visual cortex, A3, and AuA1), with males represented by blue lines and females by orange lines, showing different expression patterns over time. The graph indicates the average expression of *EOLA2* in males and females at 1M, 3M, and 6M. Statistical analysis was conducted using the Wilcoxon Rank Sum test with Bonferroni correction (**P < 0.01).

Fresh frozen brain slices (coronal plane) were sectioned (10 μm thickness) and placed on 6.5 mm × 6.5 mm, 10x Genomics Visium slides (Fig. 1a). We profiled spatial gene expression patterns of common marmoset cortices encompassing the primary and secondary visual cortex (V1 and V2, respectively), primary auditory cortex (AuA1), primary somatosensory cortex (A3), and prefrontal cortex (PFC) at different postnatal developmental ages: P16, male, 1-month (M), 3-months (M) and 6-months (M), for both sexes^11^ (Fig. 1a and Supplementary Table 1). We detected 11,368 ± 5,176 unique molecular identifiers (UMIs) per spot and 4,289 ± 1,273 unique genes per spot from all 47 samples (Supplementary Table 1). High mean rates to exonic regions of mRNAs (mean: 74.4 %) was detected compared with intronic regions (mean: 2.4 %) (Supplementary Table 1). The spots classified as white matter, and unreliable/mispositioned spots, and noise spots were excluded from further analysis because of tissue loss, damage, ambiguous clustering. The analysis revealed the molecularly distinct clusters for cortical layers (layer 1 (L1) to L6), as shown in the uniform manifold approximation and projection (UMAP) (Extended Data Fig. 1). Several resources have compiled genes that exhibit laminar-specific expression across both rodent and human cortices, which were used for analysis^12,13^. Although both overlapping and unique marker genes have been identified, these studies used different technologies, examined different developmental periods, and queried different regions of the cortex^13–15^. We thus assessed the robustness of these previously identified marker genes in our marmoset cortex layer enriched gene expression dataset. We generated aggregated layer-enriched expression profiles for each Visium data using a ‘supervised’ approach to assign individual spots to each of the six neocortical layers. The comparison between clusters of cortical layers identified genes enriched in each layer, which is displayed in the heatmap (Fig. 1a).

We next analyzed 10x Visium samples collected from each developmental period (P16, 1M, 3M, and 6M) for each brain regions. The data from each developmental periods was merged after clustering into each cortical layers at each developmental periods using the Seurat R package^16,17^. The batch correction was conducted using the Harmony R package^18^, followed by UMAP visualization for each developmental period (Extended Data Fig. 1).

First, we performed gene expression analysis to identify genes that exhibit sexually specific expression in various brain regions. Moreover, we extracted genes which are derived from sex chromosomes, and identified genes that exhibit temporal and brain region-specific expression changes, as indicated in Supplementary Table 2.

Among the analyzed brain regions, we observed strong expression of the gene *LOC118150934* in males but minimal expression in females (Fig. 1b). The gene *EOLA1* displayed a trend of opposite expression changes in male (decrease) and females (increase) (Fig. 1b). *EOLA2* exhibited different temporal expression changes between males and females in each brain regions (Fig. 1b,c). Additionally, we revealed genes such as *PCSK1N* and *TMSB4X* that exhibited specific expression changes in specific brain region (Fig. 1b). The spatial organization of sexually differentially expressed genes provides valuable insights into their potential interactions within neural circuits and their roles in brain function. By analyzing spatial transcriptomics data, we can investigate co-expression networks and identify gene regulatory networks that are specific to each sex. The observed differences in gene expression patterns between males and females may contribute to the developmental and functional disparities observed in their respective brains. For example, a recent paper showed female-specific risk genes involved in depressive-like behaviors by conducting single-cell RNA sequencing (scRNA-seq) and spatial transcriptomic data in non-human primate^19^.

### Analysis of layer specific marker genes in entire cortex

**Figure 2:**
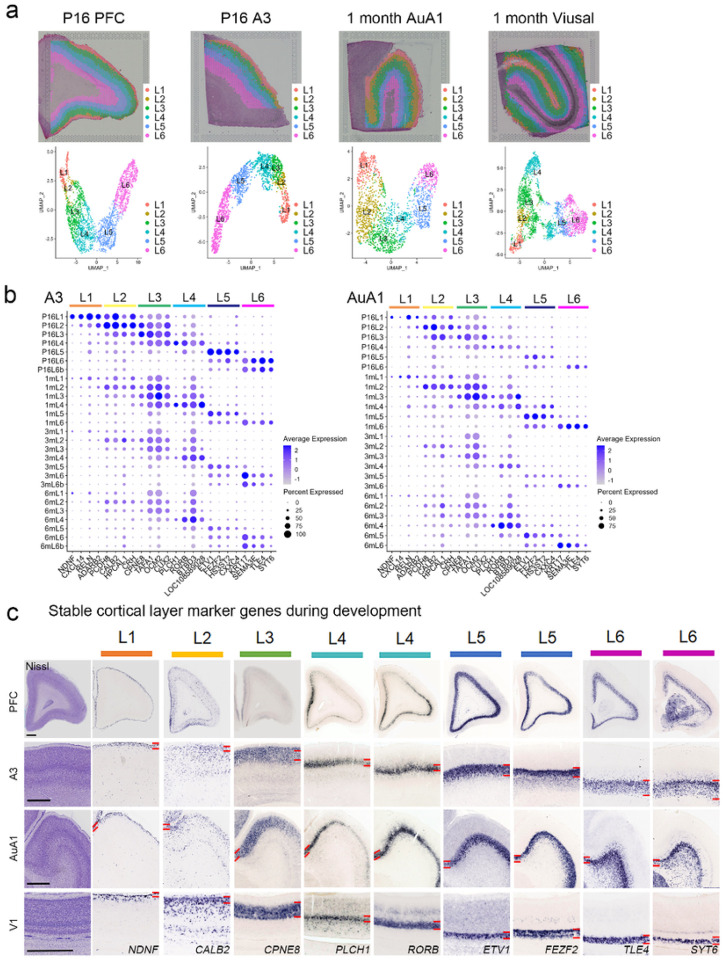
Stable cortical layer marker genes in the developing marmoset cortex (a) The clusters for the P16 PFC and A3 cortical layers (layer 1 (L1), layer 2 (L2), layer 3 (L3), layer 4 (L4), layer 5 (L5), layer 6 (L6)) were displayed in the registered spatial images (upper panels). Additionally, the clusters for 1M AuA1 and visual cortex (V1 and V2) cortical layers (L1, L2, L3, L4, L5, and L6)) were displayed in the registered spatial images (upper panels). Molecularly distinct clusters of cortical layers for PFC, A3, AuA1, and visual cortex were visualized on the UMAP (lower panels). (b) The clusters for cortical layers identified stable cortical layer marker genes in the developing marmoset cortex. The expression of stable cortical layer marker genes in each layer were shown in the dot plots for A3 (left panel) and AuA1 (right panel) during development (P16, 1M, 3M, and 6M). (c) The stable cortical layer marker genes were confirmed in the developing marmoset cortex. In situ hybridization (ISH) confirmed the specific cortical layer expression for *NDNF*(L1), *CALB2* (L2), *CPNE8* (L3), *PLCH1* (L4), *RORB* (L4), *ETV1* (L5), *FEZF2* (L5), *TLE4* (L6), and *SYT6* (L6) in PFC, A3, AuA1, and V1 at P0. Scale bars: 1 mm.

To reveal the layer specific marker genes that are stably expressed in all cortical regions across all analyzed developmental periods, we performed clustering for PFC, A3, V1, and V2 cortical layers at P16, 1M, 3M, and 6M (Fig. 2a). Major common layer markers are visualized by dot plot showing the relative expression of a subset of layer marker genes (x-axis) across all layers (y-axis) (Fig. 2b, Extended Data Fig. 2, and Supplementary Table 3). We used the marmoset gene atlas (MGA) database (https://gene-atlas.brainminds.jp/) to validate the identified cortical layer-specific markers^8,20^. *In situ* hybridization (ISH) revealed their layer specific expression patterns in the marmoset cortex at P0 (*NDNF* (layer 1), *CALB2* (layer 2), *CPNE8* (layer 3), *PLCH1* and *RORB* (layer4), *ETV1* and *FEZF2* (layer 5), *TLE4* and *SYT6* (layer 6) across 4 cortical regions (PFC, A3, AuA1 and V1) (Fig. 2c).

Together, these analyses demonstrate the power of concurrently acquiring histology and gene expression data and highlight the ability of the 10x Visium platform to achieve high-resolution spatial expression profiling within the developing marmoset neocortex.

### Identifying time dependent layer-enriched genes in marmoset visual cortex

**Figure 3:**
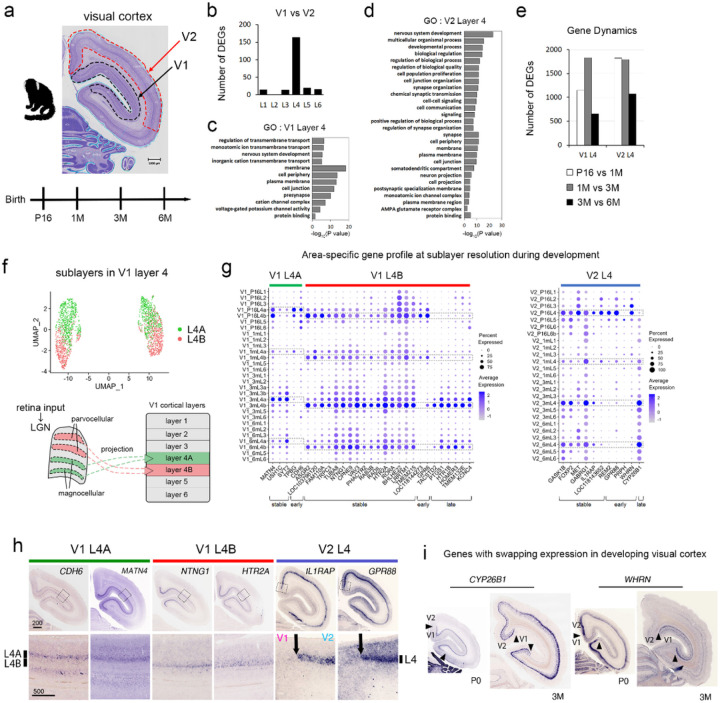
Spatiotemporal gene expression at sublayer resolution in the developing visual cortex (a) A schematic representation illustrates the time course for 10x Visium analysis of V1 (black dashed line) and V2 (red dashed line), as shown in the Nissl image. (b) The number of conserved differentially expressed genes (DEGs) was extracted from the comparison of each layer of V1 and V2 at same time period. (c, d) Gene ontology (GO) analysis from the comparison between V1 L4 and V2 L4. The graphs show GO terms associated with V1 L4 (c) and V2 L4 (d) for biological process (BP), cellular component (CC), and molecular function (MF). Common GO terms were extracted from the comparison at each time period (P16, 1M, 3M, and 6M). For V2 L4 graph (d), top 15 GO terms for BP were shown in the graph. (e) The comparison of DEGs in L4 at each period was conducted separately for V1 and V2. The graph indicates the number of DEGs between P16 L4 and 1M L4 (white bars), 1M L4 and 3M L4 (gray bars), and 3M L4 and 6M L4 (black bars) in V1 and V2, respectively. (f) A UMAP illustrates molecularly distinct clusters for V1 L4 sublayers during development (V1 L4A; green dots and V1 L4B; red dots). The schema shows that V1 L4A and V1L4B receive input from the magnocellular layers (green) and parvocellular layers (red) of lateral geniculate nucleus (LGN), respectively. (g) Dot plots illustrate stably and transiently enriched genes in V1 L4A, V1 L4B, and V2 L4 during development. (h) ISH confirmed the unique expression patterns of marker genes in visual cortex at P0 (NTNG1 and MATN4in V1 L4A, NTNG1 and HTR2A in V1 L4B, and IL1RAP and GPR88 in V2 L4) (i) Genes with swapping expression in the visual cortex during development were validated by ISH at P0 and 3M, demonstrating dynamic gene expression for *CYP26B1* and *WHRN* in a particular layer of visual cortex.

The critical period in the primate visual cortex is a crucial period characterized by significant changes in the flexibility of neurons^21^. This period is marked by specific genes undergoing functional and expression changes in response to appropriate visual stimuli, especially for the development of connections between neurons and the formation of neural circuits^22,23^. For example, V1 and V2 are distinct regions of the brain that participate in visual processing^24^. Therefore, V1 and V2 differ in terms of developmental timing, cellular specialization, connectivity, and functional integration. To comprehend these differences during development, we compared the expression profile between V1 and V2 cortices in developing marmoset (Fig. 3a).

As Fig. 2 analysis identified clusters for six cortical layers of V1 and V2 during development (Extended Data Fig. 1), we directly compared the gene expression between V1 and V2 cortical layers. Direct comparison for V1 layer 4 and V2 layer 4 clusters indicates significant gene differences during development, however, such significant gene differences were not detected when other layer clusters of V1 and V2 were compared during development (Fig. 3b). This suggests that the layer 4 is a characteristic cortical layer in visual cortex, therefore, we focused on the gene expression profiles of layer 4 in developing marmoset visual cortex. The gene ontology (GO) analysis of genes obtained from the comparison between V1 layer 4 and V2 layer 4 showed various categories such as “nervous development”, “neuron projection”, and “ion channels” (Fig. 3d,e). In general, V1 matures earlier, specializes in fundamental visual processing, and establishes specific connections with both visual and non-visual areas^25^. On the other hand, V2 develops later, demonstrates higher cellular specialization, forms more extensive connections with other visual regions, and engages in more intricate visual processing tasks^26^. Consistent with this, our comparison between V1 layer 4 and V2 layer 4 showed distinct gene expression dynamics during development. (Fig. 3e). The number of differentially expressed genes (DEGs) in V1 at different periods reached its peak after 3M and then decreased at 6M, showing the dynamic gene expression from P16 to 6M (Fig. 3e). On the other hand, V2 also exhibited dynamic changes in gene expression during the P16–3M time period, indicating that V2 undergoes dynamic plasticity during this period (Fig. 3e).

To enhance understanding of our GO analysis, we focused on the comparison between V1 layer 4 and V2 layer 4 at P16, given that gene expression, including axon guidance molecules, appears to be dynamic during early development (Supplementary Table 4). GO analysis at P16 revealed the categories for “neuron projection development” and “ion channels” in V1 and V2 layer 4, which often characterize area-, layer-, or cell-type-specificities. For example, Semaphorins which are involved in axon guidance exhibited area-, and layer-specific expression in the visual cortex (*SEMA3F, SEMA5A, SEMA5B,* and *SEMA7A* in V1, and *SEMA3A* in V2) (Supplementary Table 4). Furthermore, genes encoding ion channels exhibited unique expression patterns in the visual cortex (*KCNA2, KCNC1, KCNC2, KCNJ3, KCNQ5, KCNS1, KCNS2, SCN1B,* and *SCN2B* in V1, and *CACNG3, CACNA1I, GABRA5, GABRG1, KCNA4, KCNAB1, KCNF1*, and *KCNG1* in V2) (Supplementary Table 4). Primate V1 layer 4 is divided into two sublayers: 4A and 4B. These sublayers process visual information differently, with 4A receiving direct input from the thalamus and relaying information related to visual perception. Neurons in 4A receive inputs from pathways processing motion and color. In contrast, 4B processes orientation and spatial frequency information. Layer 4 in V2 continues processing visual stimuli with more complexity. Understanding these layers’ functional properties and connectivity patterns is crucial for understanding visual perception and higher-order visual processing. We investigated genes with spatiotemporal expression patterns at sublayer resolution, focusing on layer 4A and 4B in V1 (Fig. 3f). A direct comparison among V1 layer 4A, V1 layer 4B, and V2 layer 4 identified genes with spatiotemporal expression patterns in these layers, along with the area- and layer-specific genes that exhibit stable expression during development (Fig. 3g). ISH confirmed their stable- and area-specific expression at sublayer level in visual cortex (V1 layer 4A: *MATN4*; V1 layer 4B: *NTNG1, HTR2A;* V2 layer 4: *IL1RAP* in Fig. 3g,h). For spatiotemporal genes, *CDH6* in V1 layer 4A, *GPR6* in V1 layer 4B, and *GPR88* in V2 layer 4 exhibited transient expression at early periods (Fig. 3g,h). Finally, through these analyses, we identified certain genes, like *CYP26B1* and *WHRN*, with expression patterns that swap between V1 and V2 at specific developmental time points (Fig. 3i). This gradual specialization and maturation of visual processing in these areas, with V1 focusing on initial visual processing and V2 contributing to higher-order visual processing, may give the appearance of swapped gene expression during development.

Analyzing genes specifically expressed in V1 and V2 at sublayer resolution is crucial for comprehending the developmental processes, cellular differentiation, circuitry, connectivity, functional specialization, and neurobiology of these visual areas.

### Identification of spatiotemporally expressed genes specific to area, time, and both area and time in the marmoset PFC

**Figure 4:**
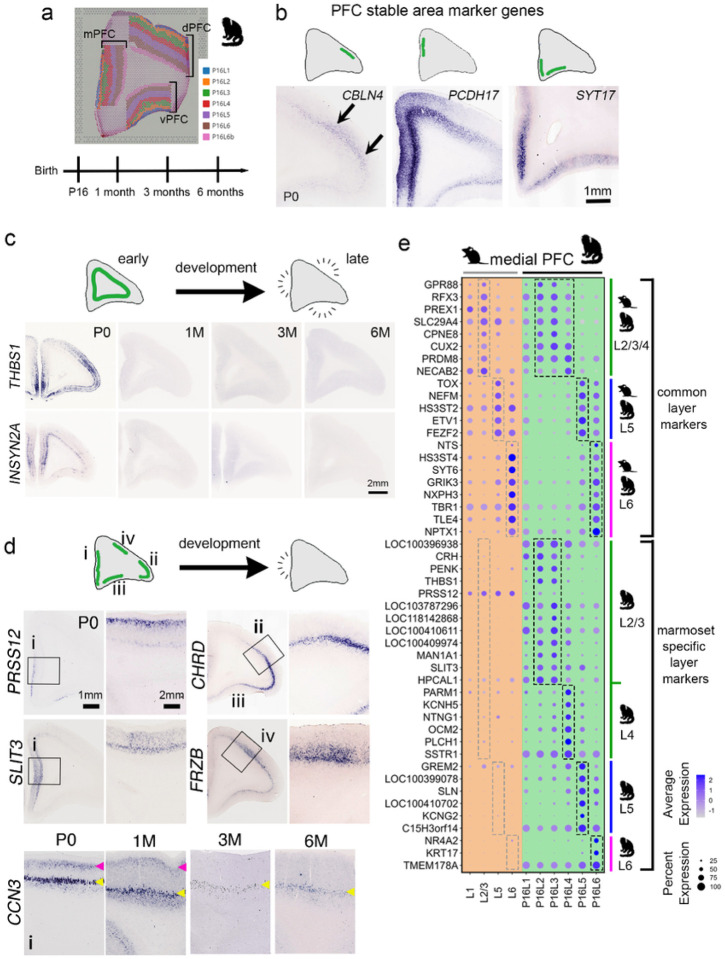
Identification of genes specific to area, time, and both area and time in the developing marmoset PFC (a) Schema for 10x Visium analysis of developing marmoset PFC. A representative image for the clusters of cortical layers for each region (mPFC, vPFC, dPFC) was presented in the registered spatial image at P16. (b) Confirmation of expression patterns for PFC stable area marker genes within PFC. ISH validated the enriched expression of area marker genes within PFC at P0 (*CBLN4* in dPFC, *PCDH17* in mPFC, and *SYT17* in vmPFC and vPFC). (c) Confirmation of expression patterns for PFC temporal marker genes within PFC. ISH for *THBS1* and *INSYN2A* validated the enriched expression at early developmental stage in the developing PFC (P0, 1M, 3M, and 6M). (d) Confirmation of expression patterns for PFC spatiotemporal marker genes within PFC. ISH for *PRSS12, SLIT3, CHRD, FRZB,* and *CCN3* validated area-, time-, layer-specific expression in developing PFC. Each PFC region is indicated in the schema (i: mPFC, ii: dlPFC, iii: vmPFC, iv: dmPFC). (e) A dot plot showed distinct cortical layer marker genes in mPFC between mice and marmosets. Comparison of mPFC layer marker genes between P7 mice and P16 marmosets identified conserved and marmoset-specific layer marker genes in the developing mPFC. The upper panel displays conserved mPFC layer marker genes between species, while the lower panel displays marmoset-specific mPFC layer marker genes.

The primate prefrontal cortex (PFC) is divided into several regions, including the dorsal prefrontal cortex (dPFC), the medial prefrontal cortex (mPFC), and the ventrolateral prefrontal cortex (vPFC) (Fig. 4a). These cortical areas are highly evolved in primate and they play distinct roles in higher-order cognitive processes and exhibit differences compared to the PFC of mice^27^. The PFC in primates possesses extensive interconnections with various cortical regions, encompassing sensory and association areas^28^. Consequently, the Identification of genes specific to area, time, and both area and time holds the potential to unveil on the mechanisms underlying the distinctive connectivity and functional development of the primate-specific PFC. To identify these marker genes, we compared layer makers in each PFC area as landmarks and identified area-specific and layer-specific genes in PFC (Supplementary Table 5). We first looked for genes which expression is stable during the development and have area specificity (Fig. 4b). Although the regional boundaries are less clear in PFC, some genes have relative regional identities (*CBLN4* in dlPFC, *PCDH17* in mPFC, and *SYT17* in vmPFC and vPFC) (Fig. 4b and Supplementary Table 5). The primate PFC is known to be a complex cortical region with distinct subregions and cytoarchitectonic areas. Identifying regional markers in Supplementary Table 5 helps to define the boundaries and organization of different PFC areas, providing a structural framework for understanding its functional organization. In addition to the area marker genes, we identified genes with temporal expression (*THBS1* and *INSYN2A*, Fig. 4c). Finally, we focused on genes that exhibited specific layer and regional identities, leading us to identify genes which showed spatiotemporal expression pattern in the marmoset PFC (Fig. 4d). *PRSS12* in mPFC and vPFC layer 2, *CHRD* in dPFC layer 5, *SLIT3* in mPFC, *FRZB* in dPFC, and *CCN3* in mPFC layer 2 exhibited transient expression during early developmental stage (Fig. 4d). We further compared the genes that show time- and region-specific expression in the PFC of marmosets with that in the prelimbic region and infralimbic region (PrL and IL) of mice to determine whether they are involved in the specificity of the PFC of marmosets. The results revealed that number of genes expression specific to marmoset (Fig. 4e).

This suggests that the PFC is a highly evolved region in primates, and differences in gene expression during development may lead to unique circuit formation and function in primates.

### Spatiotemporal gene expression controls PFC circuits

**Figure 5:**
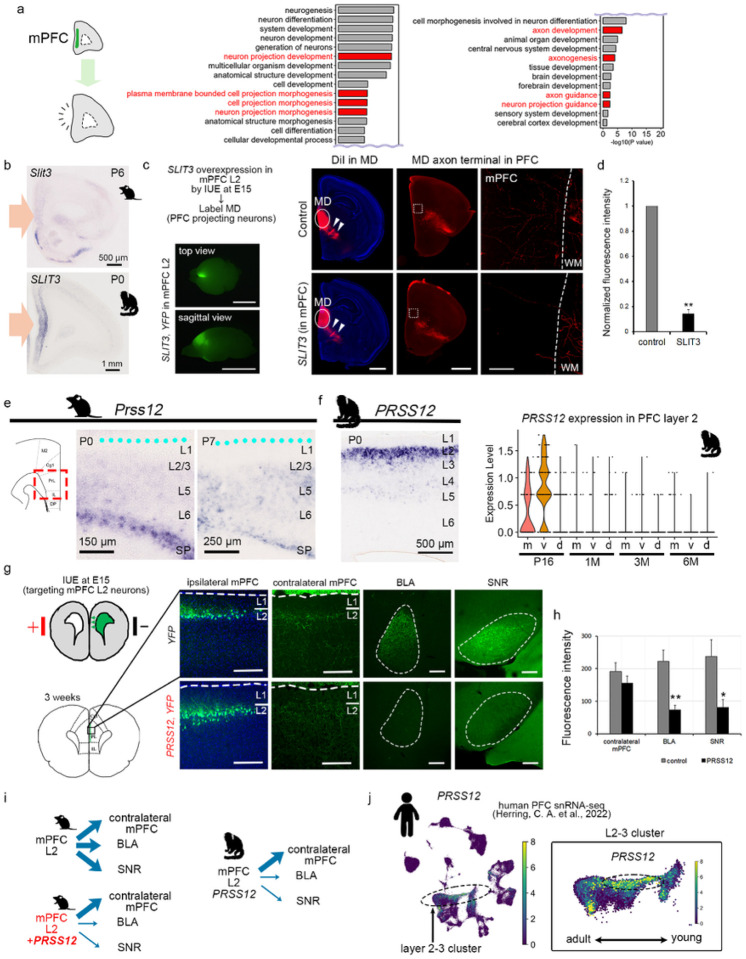
Marmoset specific genes control PFC neural circuits. (a) GO analysis from the comparison between P16 mPFC and 3M mPFC. The graphs show GO terms showing similarity to the category of “nervous system development” for biological process (BP). (b) ISH confirmed SLIT3 is highly expressed in marmoset mFPC at P0 not in mice mPFC at P6. (c) Schema of over expression of *SLIT3* in mouse mPFC L2 neurons using IUE. Brains were harvested at P6 or 7, then DiI crystal was placed on MD of hemi-dissected brains to label MD neurons. Representative images showed co-electroporated *YFP* expression in mouse mPFC. Scale bars, 5 mm. Representative images showed Dil-labelled MD axons (white arrows) in MD regions and PFC in control and *SLIT3*-overexpressed brains. Scale bars, 1 mm. The mPFC regions are shown in the high-magnification images of the white dotted boxes. The white dashed lines indicate the border between white matters and gray matters. Scale bar, 100 mm. (d) Quantitative analysis of fluorescent intensities in mPFC of control and SLIT3-overexpression brains. The values normalized with control indicate the mean ± SEM. Mann-Whitney U test, **P < 0.01. (e) ISH showed *Prss12* expression at P0 and P7 mouse mPFC. The surface of this part is marked by the blue dots. (f) ISH validated the layer 2-specific expression for *PRSS12* in the mPFC of marmoset at P0. Additionally, a violin plot showed dynamic expression for *PRSS12* during development in marmoset PFC from P16 to 6M. (g) Schema of over expression of *PRSS12* in mouse mPFC L2 neurons using IUE. Brains were harvested at 3 weeks and co-electroporated *YFP* expression in cell bodies and fibers are observed in electroporation site, contralateral mPFC, BLA, and SNR. Scale bars, 200 mm. (h) Quantitative analysis of fluorescent intensities in each region. The values indicate the mean ± SEM. Mann-Whitney U test, *P < 0.05 and **P < 0.01. (i) The cartoon depicts the projection patterns from mPFC L2 neurons to contralateral mPFC, BLA, and SNR in mice and marmoset. In mice, mPFC L2 neurons project to the contralateral mPFC, BLA, SNR in control. However, PRSS12 overexpressing mouse mPFC L2 neurons showed decreased projection to BLA and SNR. Similarly, the marmoset A32 exhibits less projection to BLA and SNR compared to contralateral mPFC. (j) The scRNA-seq data from the human PFC exhibited similar expression patterns of *PRSS12* compared to marmosets. *PRSS12* is predominantly expressed in L2–3 cluster (highlighted by a black dashed circle in the left panel) during early developmental stages (highlighted by a black dashed circle in the right panel).

To understand gene function involved during early development in the marmoset PFC, we conducted GO analysis between datasets from early developmental stage and later developmental stage of mPFC. The GO analysis revealed the presence of abundant GO terms showing similarity to the category of “nervous system development” in the mFPC at the early developmental stage (Fig. 5a). Notably, a significant number of genes that showed spatiotemporal expression in the developing marmoset PFC were discovered to hold pivotal functions in neural circuit formation, including aspects like dendritic morphology and axon guidance (Fig. 5a and Supplementary Table 6). These results strongly suggest that the primate cerebral cortex undergoes dynamic neural circuit formation in the early stages of life. This process entails the region- and layer-specific expression of genes critical for the development of neural circuits at various stages. However, it remains unclear how these spatiotemporal gene expression controls PFC development.

One of those genes, *SLIT3* was highly expressed in marmoset mPFC but not in mouse PrL in early postnatal stage P0 (Fig. 5b). As mPFC receives input from various brain regions, including contralateral mPFC, mediodorsal thalamus (MD), and amygdala, we hypothesized that SLIT3, a known repulsive guidance molecule, controls axon guidance to the mPFC. To investigate this hypothesis, we tested SLIT3 function for axon guidance from MD to mouse PrL^29^. *SLIT3* was ectopically overexpressed in mouse PrL layer 2 neurons by in utero electroporation (IUE), then MD axons was labeled by DiI placement on MD at P6 or 7 (Fig. 5c). Consistent with previous observations, MD neurons reached their axons to the mPFC in control animals (Fig. 5c). On the other hand, MD neurons failed to innervate into mPFC cortical layers when *SLIT3* was overexpressed in PrL layer 2 neurons (Fig. 5c,d). This may seem different from the mPFC-MD connection in marmosets. However, when we looked at gene expression in MD, *ROBO3*, which repels *SLIT3*, is present in mouse MD but not in marmosets (Extended Data Fig. 4). This implies that gene expression in the mPFC might have changed to match the repulsive factor in the MD. Changes in the thalamus during evolution may have influenced gene expression in the cerebral cortex or other way around.

*PRSS12* (Serine Protease 12) exhibited specific expression in layer 2 of mPFC at just one month of age, however, *Prss12* didn’t show such specific expression in the layer 2/3 of the PrL or IL regions of the developing mouse PFC (Fig. 4e–f). *PRSS12* is known as a diseases-associated gene, including Intellectual Developmental Disorder, Autosomal Recessive 1, and Autosomal Recessive Non-Syndromic Intellectual Disability^30^. Notably, mouse PrL and IL, which morphologically resemble the primate cingulate cortex, project to the contralateral cortex, amygdala, and substantia nigra (SNR) from postnatal day 10^31,32^ (Fig. 5g). In contrast, comparing the projection to region A24 (anterior cingulate cortex) in marmosets reveals a localized projection to amygdala, with no projection to SNR and the contralateral cortex. This illustrates distinct circuitry between mice and marmosets (Fig. 5d and Extended Data Fig. 3). In the marmoset PFC, projections from region A25 (subgenual cingulate area) extend significantly to the contralateral cortex and amygdala, but not to the SNR (Extended Data Fig. 3). Consequently, when the Prss12 gene, which is not specific to the mouse PrL, was overexpressed in the mouse PrL layer 2 through IUE, it resulted in reduced projections to amygdala and SNR, without altering projections to the contralateral cortex (Fig. 5g,h). Remarkably, this projection pattern mirrors that of marmoset A32, where *PRSS12* is expressed in a temporally specific manner: projection to contralateral cortex, amygdala (small projection) and SNR (small projection) (Fig. 5g–i, Extended Data Fig. 3). The homology between mouse PrL and IL and any primate brain region remains a complex question. Introducing a single *PRSS12* gene to the mouse PrL, however, results in a connection pattern resembling marmoset A32. Finally, to investigate the similarity of the marmoset and human developing PFC, we examined the expression patterns of the human *PRSS12* gene in the developing human PFC^33^. We found that PRSS12 is strongly expressed during early developmental stage in layer 2/3 neurons (Fig. 5j), which suggests that gene expression in the marmoset brain is similar to that in humans in many ways, and that the development of the marmoset brain is similar to that of humans. Understanding how genes are expressed in the developing marmoset brain will help us understand how the human brain develops and, in turn, the causes of developmental disorders.

## Discussion

To advance the scientific understanding of cortical development and function, it is essential to identify precise molecular markers exhibiting activity within specific temporal periods and regions. A comprehensive analysis using spatial transcriptomics identified sexually, spatially, and temporally differentially expressed genes in the developing marmoset cortex. These results provide valuable insights into evolutionary perspectives and contribute to our understanding of the mechanisms underlying evolution, primate development, and developmental disorders.

Temporal marker genes expressed in specific cortical layers are significant for understanding the dynamic processes of brain development and the establishment of functional circuits. The primate brain undergoes intricate developmental processes characterized by the sequential generation and maturation of different cell types and the establishment of neural circuits. Temporally expressed layer marker genes provide insights into the processes underlying circuit formation and plasticity during development, including synapse formation, neural connectivity, and synaptic plasticity, which are essential for establishing and refining functional circuits. Comparing temporally expressed layer marker genes between species can shed light on the evolutionary modifications that have shaped cortical development and function in different primate lineages, contributing to our understanding of the evolutionary origins and adaptations of the primate brain.

The results of our study highlight the intricate interplay between gene expression patterns in the marmoset cortex and their implications for biological and evolutionary processes. Through a comparative analysis with mouse and marmoset, we uncovered distinct expression profiles of key genes in different species.

Notably, the temporal expression profiles of several genes in marmoset mPFC differed significantly from those in mice. These differences in gene expression are likely important for marmoset-specific circuit formation. For example, forced expression of Slit3 in mice inhibited projection from the MD. This is because the MD in mice has the receptor for Slit3 (Robo3), while the marmoset MD does not (Extended Data Fig. 4). This difference in gene expression suggests that not only the mPFC but also the thalamus in marmosets has its own gene expression pattern, leading to more species-specific neural circuits. On the other hand, *PRSS12* in the marmoset PFC, particularly its early localization to layer 2 and uncertain expression in PrL and IL regions in newborn mice, demonstrate intricate developmental variations. Interestingly, our observation that overexpression of *PRSS12* in mouse PrL layer 2 led to reduced projections to the amygdala and SNR from PrL layer 2, akin to marmoset A32’s projection pattern, suggests that specific gene expression dynamics play a pivotal role in shaping primate-specific circuits. Despite these valuable insights, the precise homology between mouse PrL and IL and their counterparts in primate brain regions remains a complex query, warranting further investigation. Nevertheless, our study underscores the intricate relationship between gene expression dynamics, brain region specificity, and timing in the establishment of species-specific neural circuits within the prefrontal cortex.

The 10x Visium technology enables high spatial resolution mapping of gene expression patterns within tissue sections, allowing comprehensive transcriptomic profiling of thousands of genes in a single sample. This technology preserves tissue morphology, facilitating histological examination or imaging alongside gene expression data. Analyzing and interpreting the large datasets generated by 10x Visium spatial transcriptomics requires specialized bioinformatics tools and expertise. To facilitate easy data access and analysis, a database has been created (accessible at https://gene-atlas.brainminds.jp/gene-visium/, pass word: MMTD@7267, pass word will be removed after the acceptance) that offers visualization of the generated data. Recently, database of mouse and macaque spatial transcriptome data has been developed to allow comparison of gene expression patterns in different species^34,35^. These publicly available resources will greatly contribute to our understanding of the mechanisms of brain evolution, primate-specific neural circuit formation and brain function, and the mechanisms of human brain pathology.

## Method

### Animal

All procedures in marmoset and mice were performed in accordance with protocols approved by the Institutional Animal Care and Use Committee of RIKEN Wako branch (W2022-2-026 and W2022-2-027). Marmosets were derived from a breeding colony at RIKEN Center for Brain Science. ICR (CD1) timed pregnant mice were purchased from Japan SLC.

### Images for marmosets

*In situ* hybridization and Nissl images were acquired from Marmoset Gene Atlas website (https://gene-atlas.brainminds.jp/). Images for tracer injection of marmoset brain were acquired from Marmoset PFC Connectome website (https://dataportal.brainminds.jp/marmoset-tracer-injection).

### Sample preparation and experimental procedure for 10x Visium

Mice were deeply anesthetized with a lethal dose of Secobarbital (150 mg/kg, i.p.). The brain placed was immediately frozen in OCT compound (Sakura Finetech). Marmosets were deeply anesthetized with a lethal dose of mixed anesthesia (0.4 mg/kg medetomidine, 2 mg/kg midazolam, and 5 mg/kg butorphanol, i.p.), followed by Secobarbital (100 mg/kg, i.p.). The brain was removed and placed in ice-cold phosphate-buffered saline (PBS). Then, the brain was immediately coronally cut into 3–4 mm thick slices, and the coronal brain slices were immediately frozen in OCT compound (Sakura Finetech). Coronal cryosections of 10 mm thickness (CM1950, Leica, RRID:SCR_018061 and HM525NX, Thermo Fisher Scientific) were mounted on Visium Tissue Optimization Slides (PN-1000192, 10x) and Visium Spatial Gene Expression Slides (PN-1000185, 10x, RRID:SCR_023571) following to the company’s instructions. The subsequent experimental work was conducted by KOTAI biotechnologies (Osaka, Japan). The detail experimental conditions and results are summarized in Supplementary Table1. In brief, Visium Spatial Tissue Optimization Reagent Kit (PN-1000192, 10x) was used for tissue optimization, and Visium Spatial Gene Expression Reagent Kit (PN-1000186, 10x), Library Construction Kit (PN-1000215, 10x), and Dual Index Kit TT Set A (PN-1000215, 10x) were used for gene expression analysis (GEX slide). Total RNA was extracted using RNeasy Mini Kit (QIAGEN), and RNA Integrity Number (RIN) was calculated using Tapestation (Agilent). For sequencing, the Novaseq S4 on NovaSeq 6000 (Illumina) was used. Raw FASTQ files and staining images were processed by Space Ranger software version 1.3.1, with Callithrix_jacchus_1700 (mCalJac4) or mm10–2020-A as the reference genome.

### Data processing for 10x Visium

For area definition, AuA1, A3, dPFC, mPFC, and dPFC areas were identified based on H&E staining, while V1 and V2 areas were determined using area-marker genes and H&E staining. After selecting each area, the data was analyzed using the Seurat package in R^16,17^. The following functions in Seurat package were utilized: *SCTransform* function for normalization, *RunPCA* function for dimensional reduction, *FindNeighbors* function for computation of the nearest neighbors, and *FindClusters* function for determining clusters.

The unsupervised clustering of the cortex into cortical layers was determined based on characteristic cortical layer markers and the position of H&E staining pattern. Manual removal of unreliable spots from the clusters, such as empty spots and misplaced spots was performed. Additionally, overlapped, and crumpled tissue were excluded from the analysis, as these spots were often associated with unreliable clusters resulting from unsupervised clustering. When merging the data, batch effect correction was conducted using Harmony^18^.

For mouse mPFC analysis, mPFC including PrL and IL were selected by H&E staining patterns. Then, similar analysis described above using Seurat identified the clusters for cortical layers (L1, 2/3, L5, and L6). The marker genes for each cluster were determined using the *FindAllMarkers* function in Seurat.

### Analysis

Cluster information obtained from the Seurat analysis was exported into Loup Browser software (10x, RRID: SCR_018555) to identify characteristic layer marker genes within the cortical layers. The top 100 up-regulated genes in each layer’s cluster were ordered based on adjusted p-values using Benjamini-Hochberg correction. From these genes, we extracted common genes across the datasets of each area and each developmental period. These common genes were defined as layer marker genes for each area at each developmental period. For definition of temporal layer marker genes for each area, overlapping marker genes during developmental period were defined as stable layer marker genes for the area, representing layer-specific expression patterns throughout development. On the other hand, non-overlapping marker genes at each developmental period were defined as temporal layer marker genes for the area, representing layer-specific expression patterns to certain developmental periods within the area. Within PFC, stable and temporal layer marker genes for mPFC, vPFC, and dPFC were compared between these areas to identify spatiotemporal layer marker genes, representing area-, time- and layer-specific expression patterns within PFC. From the above analysis, the expression was manually validated by ISH using MGA.

For visual cortex (V1 and V2), to identify differentially expressed genes (DEGs), layer clusters within two developmental periods (P16 vs 1M, 1M vs 3M, and 3M vs 6M) within the same area were compared using the *FindMarkers* function in Seurat. The comparison was conducted using Wilcoxon Rank Sum test, with a log fold change threshold greater than 0.3. The same comparison was also performed between V1 and V2 comparison during development. After comparison, gene ontology (GO) analysis for biological process (BP), cellular component (CC), and molecular function (MF) were done using R package gProfiler2 and REVIGO website (http://revigo.irb.hr/)^36,37^. For mPFC comparison during development, we compared the gene expression between P16 mPFC and 3M mPFC datasets including all cortical layers, then GO analysis using gProfiler and REVIGO identified the categories showing similarity to the category of “nervous system development” for BP.

For the analysis of sex differences, datasets for male and female PFC, A3, AuA1, and visual cortex were used for comparison (Supplementary Table 2). Entire Visium data including gray matter and white matter for male and female at each developmental stage (1M, 3M, and 6M) were compared using *FindMarkers* function. The comparison was conducted using Wilcoxon Rank Sum test, with a log fold change threshold greater than 0.25. Subsequently, differentially expressed genes between male and female derived from sex chromosomes were extracted. After merging all data for each area, average expression for each developmental stage was used for line graph. Also, their expression patterns were shown in violin plot.

For the plots, ggplot2 R package was utilized^38^.

### Single-nucleus RNA sequencing (snRNA-seq) data

To investigate the expression pattern of *PRSS12* in the human developing PFC, snRNA-seq data was obtained from publicly available dataset for the developing human PFC^33^. The L2/3-CUX2 cluster annotated in the original paper was extracted from the dataset to obtain the expression in the L2/3-CUX2 cluster during development (Fetal, Neonatal, Infancy, Childhood, Adolescence, and Adult defined in the original paper).

### *In situ* hybridization (ISH) for mouse

ISH was performed as described previously^39^. In brief, mice were deeply anesthetized with a lethal dose of pentobarbitone (150 mg/kg, i.p.) and transcardially perfused with 4% paraformaldehyde (PFA) in phosphate-buffered saline (PBS). Brains were removed and postfixed in the same fixative for 1 h at 4°C and 2 h at room temperature and equilibrated with 30% sucrose in 4% PFA at 4°C overnight. Coronal brain sections of 28 μm thickness were generated with a sliding microtome (SM2020R, Leica). Antisense probes for *Prss12, Slit3*, and *Tacr3* were generated from the template plasmids (FANTOM clone, 4833438J08 for *Prss12*, M5C1008N08 for *Slit3*, and B230383K19 for *Tacr3*). The expression patterns of each gene were validated in three independent brains.

### *In utero* electroporation (IUE)

IUE was performed as described previously^40^. pCAGGS-EYFP, pCMV3-PRSS12 (HG22620-UT, SinoBiological), or pCMV3-SLIT3 (HG17632-UT, SinoBiological) plasmid DNAs were used at E15.5 embryos. Pregnant mice were deeply anesthetized with mixed anesthesia (0.03 mg/ml, 0.4 mg/ml midazolam, and 0.5 mg/ml butorphanol, i.p.). Electric pulses were delivered with needle electrodes by an electroporator (CUY21, BEX). Three 20 V pulses of 100 ms duration were applied at intervals of 950 ms.

### Data acquisition for confocal imaging and quantification

Mice were deeply anesthetized with a lethal dose of Secobarbital (150 mg/kg, i.p.) and transcardially perfused with 4% PFA in PBS. Subsequently, the brains were removed, postfixed in the same fixative overnight at 4°C and equilibrated with 30% sucrose in PBS at 4°C overnight. For DiI labeling, the brains were removed, postfixed in the same fixative overnight at 4°C. DiI crystal (1, 1’-Dioctadecyl-3, 3, 3’, 3’-Tetramethylindocarbocyanine Perchlorate) (D282, Molecular probe) were put into mediodorsal thalamus (MD) of hemi-dissected brains under microscopy, then the brains were incubated with 1% PFA in PBS at 37°C for 12 days. Coronal brain sections (100 μm thickness) were generated using a vibratome (VT1000 S, Leica, RRID:SCR_016495). These sections were counterstained with 1 μg/mL of 4′,6-diamidino-2-phenylindole (DAPI) (11034–56, Nacalai Tesque). Fluorescent images were captured using a confocal microscopy (FV3000, Olympus, RRID:SCR_017015) or a fluorescence microscopy (BZ-X810, Keyence). The images were imported into Fiji (NIH, RRID:SCR_002285) for the analysis. For the quantification of PRSS12 experiments, the average intensity of YPF fluorescence was measured for contralateral mPFC layer 2, BLA, and SNR. The background signal intensity of YFP fluorescence from adjacent contralateral mPFC layer 1 area, where mPFC layer 2 neurons do not project, was subtracted. Likewise, the background signal intensity of YFP fluorescence from adjacent area of BLA and SNR was subtracted. The data were obtained from three independent brains for both control and *PRSS12*-overexpressed conditions. For *SLIT3* experiments, the average intensity of DiI fluorescence was measured for cortical layers and white matter in mPFC. The signal of cortical layers was normalized with the signal in white matter. The data were obtained from at least three independent brains for both control and *SLIT3*-overexpressed conditions.

## Data Availability

Spatial transcriptomics data have been deposited in the CBS repository system (https://neurodata.riken.jp/r/Shimogori/Onishi%20et%20al.,%202024/, pass word:MMBD-visium) and are publicly available as of the date of publication.
